# Central pancreatectomy in portal annular pancreas for metastatic renal cell carcinoma: a case report

**DOI:** 10.1186/s12957-019-1622-8

**Published:** 2019-04-30

**Authors:** Hiroshi Yamaguchi, Yasutoshi Kimura, Minoru Nagayama, Masafumi Imamura, Shingo Tanaka, Makoto Yoshida, Eiji Yoshida, Hiroki Fujino, Takashi Machiki, Koji Miyanishi, Toru Mizuguchi, Junji Kato, Ichiro Takemasa

**Affiliations:** 1 Department of Surgery, Surgical Oncology and Science, Sapporo Medical University School of Medicine, South-1, West-16, Sapporo, Hokkaido 060-8543 Japan; 2 Department of Medical Oncology, Sapporo Medical University School of Medicine, Sapporo, Japan; 3 Sapporo Medical University School of Health Science, Sapporo, Japan

**Keywords:** Central pancreatectomy, Circumportal pancreas, Metastatic pancreatic cancer, Portal annular pancreas, Renal cell carcinoma

## Abstract

**Background:**

Portal annular pancreas (PAP) is a rare congenital anatomical abnormality of the pancreas in which the portal vein is encircled by aberrant parenchyma, and special attention is needed for pancreatic resections. This is the first report of central pancreatectomy (CP) in a PAP for metastatic renal cell carcinoma (RCC).

**Case presentation:**

A 76-year-old man who had a history of left nephrectomy for renal cancer not otherwise specified 36 years earlier and radical cystectomy for bladder cancer 4 years earlier was incidentally found to have a pancreatic tumor and a liver tumor. The pancreatic tumor was diagnosed as metastasis of clear cell RCC, and the liver tumor was diagnosed as moderately differentiated hepatocellular carcinoma (HCC) on preoperative histological evaluation. Preoperative computed tomography imaging showed a type 3A PAP, in which the main pancreatic duct (MPD) ran ventral to the portal vein (anteportal type), and the aberrant parenchyma was located cranial to the confluence of the portal vein and splenic vein (suprasplenic type). After adhesiotomy and partial liver resection, CP was performed. With intraoperative ultrasound guidance, the aberrant parenchyma of the PAP could be preserved, avoiding additional resection. Thus, two pancreatic transections were performed, creating a single-cut margin that contained the MPD in the distal pancreas. Oncologically safe margins were confirmed by intraoperative pathological diagnosis. The distal pancreas was reconstructed by pancreatojejunostomy in the routine procedures. The pathological diagnosis of the surgical specimens was identical to the preoperative diagnosis. A postoperative pancreatic fistula (POPF) developed from the proximal stump of the head of the pancreas, necessitating no specific treatment other than drainage. The patient showed no signs or symptoms of recurrent RCC or abnormal pancreatic function for 2 years after the operation, although a histologically proven new HCC lesion developed distant from the initial site 8 months after the operation.

**Conclusions:**

Precise preoperative evaluation of the tumor features and PAP allowed adequate surgical strategies to be planned. Intraoperative ultrasound was useful to minimize parenchymal resections of the PAP. CP is still a challenging procedure in terms of the development of POPF.

## Background

Portal annular pancreas (PAP), which is also called circumportal pancreas, is a rare congenital anatomical abnormality of the pancreas. There is continuity between the uncinate process of the pancreas and the body of the pancreas via the aberrant parenchyma, resulting in the portal vein being encircled by pancreatic parenchyma. PAP is usually asymptomatic, but special attention is needed for pancreatic surgery in terms of the location of the main pancreatic duct (MPD) and the way to resect parenchyma to minimize the risk of postoperative pancreatic fistula (POPF). PAP is classified based on the running patterns of the MPD [1] and the location of the aberrant parenchyma against the confluence of the portal vein (PV) and the splenic vein (SPV) [2]. The case of a patient who had metastatic renal cell carcinoma (RCC) in the head of the pancreas with type 3A PAP and underwent central pancreatectomy (CP) is reported.

## Case presentation

**Fig. 1:**
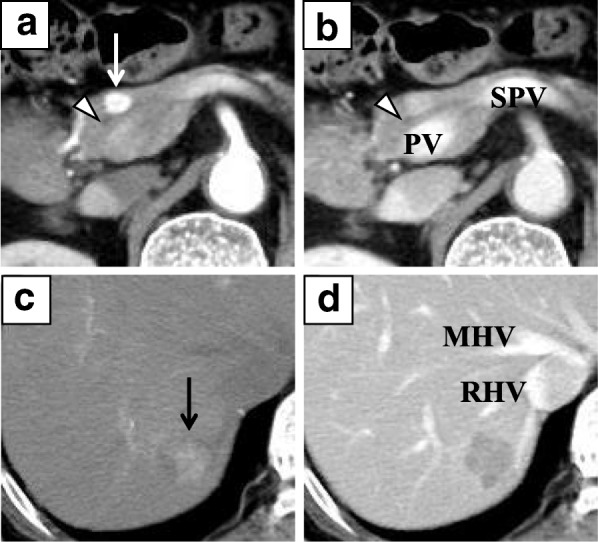
Dynamic CT findings of the pancreatic and liver tumors. **a** Arterial phase. The pancreatic tumor (white arrow) ventral to the portal vein shows strong enhancement. **b** Portal phase. The enhancement of the pancreatic tumor is attenuated. Of note, the main pancreatic duct (white arrowhead) is adjacent to the tumor. **c** Arterial phase. The liver tumor (black arrow) in Couinaud’s segment 7 shows enhancement. **d** Portal phase. The enhancement of the liver tumor washes out. PV: portal vein, SPV: splenic vein, MHV: middle hepatic vein, RHV: right hepatic vein

**Fig. 2:**
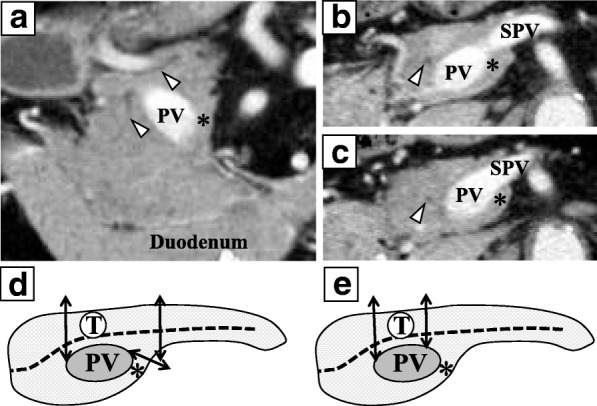
CT findings of portal annular pancreas and operative procedures. **a** Coronal image. The portal vein is completely encircled by the aberrant parenchyma of the portal annular pancreas (black asterisk). **b**, **c** Horizontal images. The aberrant parenchyma (black asterisk) is cranial to the confluence of the portal vein and the splenic vein. **c** The confluence of the portal vein and splenic vein, and **b** 5-mm cranial to **c**. Of note, the main pancreatic duct (white arrowhead) in **a** to **c** runs ventral to the portal vein toward the duodenum. **d** Preoperative simulation. Three pancreatic transections including additional resection of the aberrant parenchyma (black asterisk) are needed to remove the tumor and create the single cut margin containing the main pancreatic duct in the distal pancreas. **e** Actual operative procedure performed. The tumor is removed by two pancreatic transections preserving the aberrant parenchyma (black asterisk). The dotted line indicates the main pancreatic duct (**d**, **e**). PV: portal vein, SPV: splenic vein, T: tumor

A 76-year-old man with a history of left nephrectomy for renal cancer not otherwise specified (NOS) 36 years earlier and radical cystectomy with creation of a right cutaneous ureterostomy for invasive urothelial carcinoma of the bladder 4 years earlier was incidentally found to have a pancreatic tumor and a liver tumor on regular follow-up computed tomography (CT) after radical surgery for bladder cancer. On dynamic CT, the pancreatic tumor was located in the head of the pancreas, ventral to the portal vein, with a size of 10 mm, and it showed clear, strong enhancement in the arterial phase (Fig. 1a, b). The liver tumor was located in Couinaud’s liver segment 7, with a size of 22 mm, and it showed enhancement in the arterial phase and wash-out in the portal phase (Fig. 1c, d). No abnormal accumulation was detected in the systemic organs on ^18^F-fluorodeoxyglucose-positron emission tomography (FDG-PET). FDG-PET was negative for the pancreatic and liver tumors. To identify tumor features, endoscopic ultrasound-guided fine-needle aspiration (EUS-FUA) for the pancreatic tumor and percutaneous ultrasound-guided biopsy for the liver tumor were performed. Histologically, the pancreatic tumor was diagnosed as metastasis of clear cell RCC, with positive staining for CD10 and vimentin and negative staining for CK7, CK20, alpha-fetoprotein, and neuroendocrine markers on immunohistochemical analysis. The liver tumor was diagnosed as moderately differentiated hepatocellular carcinoma (HCC). Preoperative CT imaging also showed type 3A PAP, in which the MPD ran ventral to the portal vein, and the aberrant parenchyma was located cranial to the confluence of the PV and SPV (Fig. 2a–c). The pancreatic tumor contacted the MPD, and partial pancreatectomy was avoided to prevent injury to the MPD (Fig. 1a, b). CP with additional stapler resection and closure of the aberrant parenchyma, needing a total of three pancreatic transections (Fig. 2d), was planned.

**Fig. 3:**
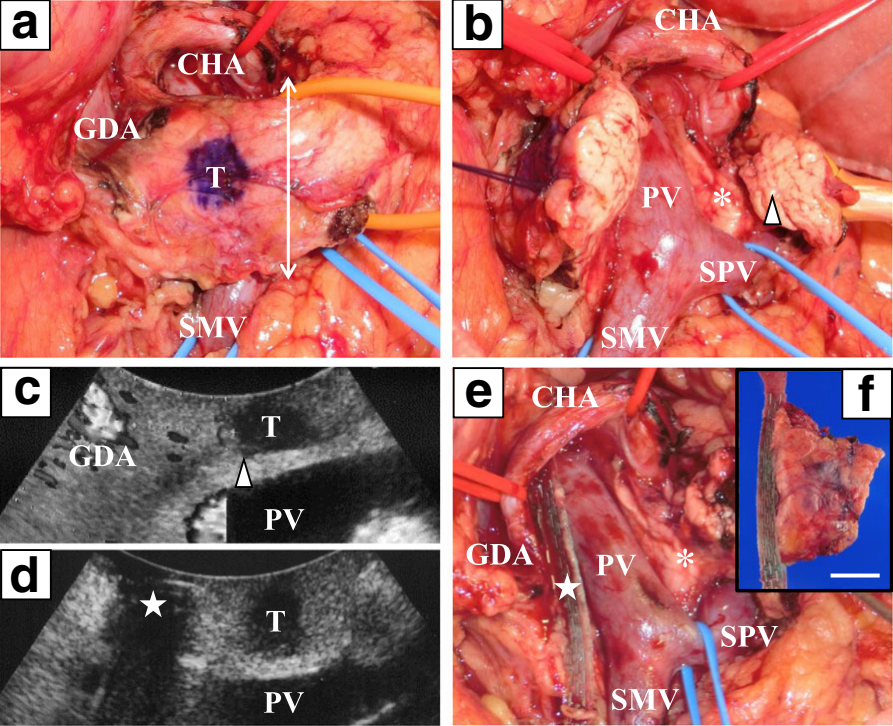
Surgical findings. **a** The location of the tumor identified by intraoperative ultrasound is marked on the surface of the mobilized pancreas. The white double-arrowed line indicates the transection line of the distal pancreas. **b** After the first pancreatic transection at the distal side, the aberrant parenchyma of the portal annular pancreas between the uncinate process and body (white asterisk) is confirmed cranial to the confluence of the portal vein and splenic vein. Of note, the single-cut margin containing the main pancreatic duct (white arrowhead) is created at the distal pancreas. **c**, **d** Intraoperative ultrasound clearly shows the pancreatic tumor (white arrowhead) giving the information of the second transection line at the pancreatic head (white star). The main pancreatic duct, which is negative for a color Doppler signal, is also clearly seen (white arrowhead in **c**). **e** After the second pancreatic transection, the tumor is removed. **f** The surgical specimen. White bar is 1 cm. CHA: common hepatic artery, GDA: gastroduodenal artery, PV: portal vein, SMV: superior mesenteric vein, SPV: splenic vein, T: tumor

After the adhesiotomy and partial liver resection, the superior mesenteric vein (SMV), SPV, common hepatic artery (CHA), and gastroduodenal artery (GDA) were well mobilized from the head of the pancreas. Then, tunneling of the pancreatic neck over the PV was completed, and the neck and body of the pancreas were well mobilized from the PV and SPV (Fig. 3a). Intraoperative ultrasound showed the tumor in the head of the pancreas and the MPD clearly, allowing precise determination of the safe surgical margins (Fig. 3c). The margin of the pancreatic tail side was first cut using a cold scalpel, and the MPD was identified in the cut surface. Intraoperative findings were consistent with type 3A PAP (Fig. 3b). The safe margin on the pancreatic head side was also determined by ultrasound and compressed by a bowel clamp for 5 min before 10-min stapler resection and closure (Fig. 3d, e). Finally, the specimen (Fig. 3f) was removed from the pancreas by two ultrasound-guided pancreatic transections, avoiding the additional resection of the aberrant parenchyma (Fig. 2e). Negative surgical margins were confirmed by intraoperative pathological frozen section diagnosis. The distal pancreas was reconstructed by the pancreatojejunostomy (PJ) applying a type of Blumgart method [3]. The operative time including adhesiotomy and partial liver resection was 522 min, and blood loss was 270 mL.

**Fig. 4:**
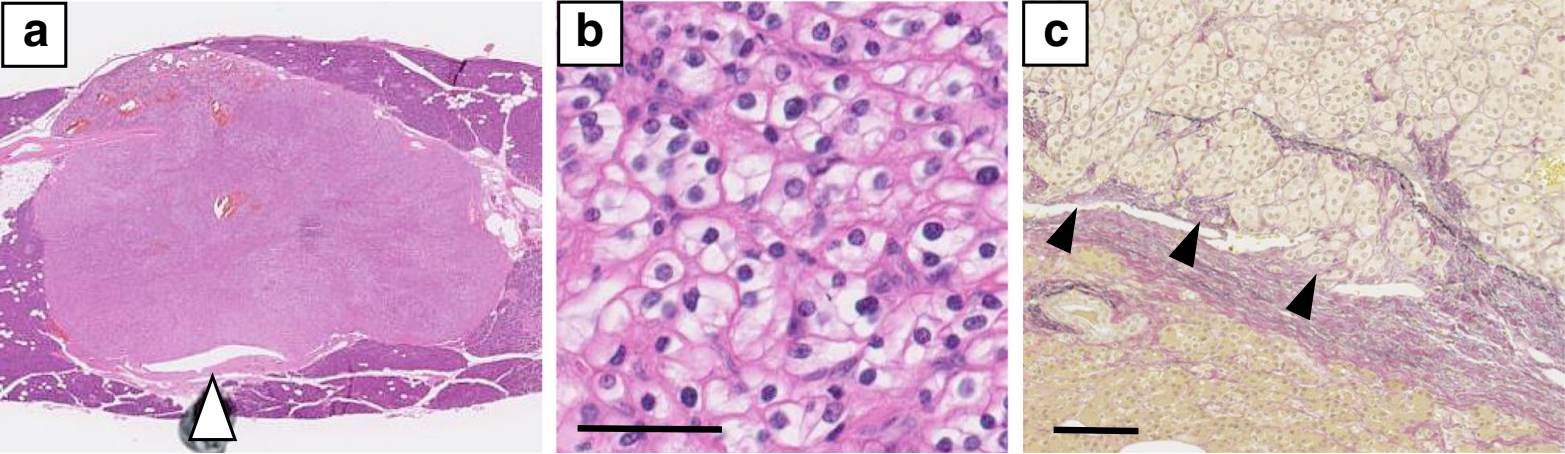
Pathological findings of the pancreatic tumor. **a** Overview of the tumor (hematoxylin and eosin staining). The tumor shows a clear margin with normal pancreatic tissue. Of note, the tumor completely contacts the main pancreatic duct (white arrowhead). **b** High magnification of **a**. Tumor cells show clear cytoplasm, diagnosed as clear cell renal cell carcinoma. Black bar is 50 μm. **c** Elastica van Gieson staining. The tumor has a fibrous capsule showing capsular invasion (black arrowheads). Black bar is 100 μm

The final pathological diagnosis was identical to the preoperative diagnosis. Histologically, the pancreatic metastasis of clear cell RCC that contacted the MPD formed a fibrous capsule and showed capsular invasion (Fig. 4). The proximal cephalic margin without the stapler was 5 mm, and the distal tail-side margin was 9 mm from the tumor. The patient was treated postoperatively for an ISGPS Grade B pancreatic fistula [4] from the stapler-closed cephalic stump (Fig. 3e). No specific treatment other than drainage was needed for the POPF, and the patient was discharged 38 days after the operation. Close follow-up without adjuvant treatment was continued due to repeated pyelonephritis. Although a histologically proven new HCC lesion developed distant from the initial site 8 months after the operation, the patient continued the treatment for HCC and showed no signs and symptoms of the recurrent RCC or abnormal pancreatic function for 2 years after the operation. Genetic analysis was not performed at the patient’s request.

## Discussion

PAP is a rare congenital anatomical abnormality of the pancreas that is usually asymptomatic. Analysis of CT imaging showed a prevalence of 0.8 to 2.5% [2, 5, 6]. In recent actual surgical cases, PAP was confirmed in 0.5 to 1.7% of patients who underwent pancreatic resections [7–9]. PAP is classified depending on the running patterns of the MPD (types 1 to 3 [1]) and the location of the aberrant parenchyma against the confluence of the PV and the SPV [2], which are well summarized in the previous reports [8, 10]. In the present case, preoperative and intraoperative findings were consistent with type 3A, in which the MPD was located ventral to the PV (anteportal type), and the aberrant parenchyma was located cranial to the SPV (suprasplenic type). Type 3A is the most common classification, accounting for 44.4% of reported cases [10] or 92% of CT-diagnosed cases [6]. Special attention is needed for pancreatic resection in PAP, especially with the development of POPF, implying a risk of modified resections or an increased number of cut margins for the aberrant parenchyma. In the review articles, POPF developed in 46.7% [10] or 38.5% [11] of analyzed PAP cases. Controversial results have been reported from surgical case series. The rate of POPF grade B or C in PAP cases was recorded as 44% after pancreaticoduodenectomy (PD) and distal pancreatectomy (DP) or 71.4% after PD, showing a significant difference from the corresponding rate in the cases without PAP, 14% or 31.7%, respectively [7, 9]. In contrast, Luu et al. [8] reported no POPF in PD cases with PAP who underwent additional resection to remove the aberrant parenchyma of PAP and create the single-cut margin containing the MPD. They also performed total pancreatectomy to avoid high-risk reconstruction. Pardiwala et al. [12] discussed the usefulness of intraoperative ultrasound to determine the anatomy of the pancreatic ducts and the importance of adequate preoperative and intraoperative planning to minimize postoperative complications in PAP. In the present case, precise preoperative diagnosis of the tumor features and the PAP, including the location of the MPD, led to a well-planned surgical strategy.

To the best of our knowledge, this is the first report of CP in PAP. Intraoperative ultrasound was also useful to determine oncologically safe surgical margins and minimize the parenchymal resection, preserving the aberrant parenchyma of the PAP during CP. The patient did not show any signs or symptoms of abnormal pancreatic function for 2 years after the operation. A single-cut margin containing the MPD in the distal pancreas was created, and no changes in the routine procedures were needed for PJ reconstruction. However, POPF grade B developed from the stapler-closed cephalic stump. Sho et al. [13] reported that double PJ with reconstruction of the MPDs in both the cephalic and distal stumps might be effective for reducing POPF, suggesting the importance of decompression of the pancreatic ducts in the head of the pancreas. On the other hand, PJ for the stump during DP did not significantly reduce POPF compared with stapler closure in a prospective, randomized controlled trial [14]. CP is still a challenging procedure providing better pancreatic function and a higher POPF rate than major pancreatic resections [15–17]. Recently, Yang et al. [18] reported encouraging results from external drainage of monolayer PJ for the distal pancreas and stapler closure of the cephalic stump in CP showing 12.1% grade B POPF and no grade C POPF, reoperation or mortality. Further studies are warranted to reduce POPF after CP.

Pancreatic metastasis (PMet) from other primary cancers is rare, accounting for about 1–2% of all pancreatic cancers [19, 20]. It has been reported that the benefit of pancreatic resections is linked to the biology of the primary cancers, suggesting the importance of precise histological diagnosis of pancreatic lesions to determine treatment strategies [20]. The most common primary cancer of PMet is RCC, in both operative and EUS-FNA-diagnosed cases [20, 21]. A previous review article [22] suggested the benefit of pancreatic resections in metastatic RCC, although the in-hospital mortality rate after pancreatic surgery for RCC was 2.8%. CP was indicated for a solitary PMet near the neck of the pancreas to preserve pancreatic function, avoiding major pancreatectomies [23]. We previously reported that some PMets of RCC (15.6%) developed extracapsular invasion with a distance from 100 to 250 μm, attracting attention to the oncological safety of surgical margins in limited resections [24]. PMets of RCC could arise in a synchronous or a metachronous manner. The median interval between nephrectomy and pancreatic recurrence was 104 months (range 0–348 months) [19]. Although the patient had a history of left nephrectomy for renal cancer NOS 36 years earlier, this case was extremely rare as a recurrence of RCC. Some cases of metastatic RCC without evidence of a primary renal tumor have been reported [25–28]. It is difficult to exclude the possibility of a small occult lesion in the remnant right kidney of the present case as a primary site of RCC, although no abnormal CT findings were observed in the right kidney for 2 years after the operation. This patient requires ongoing close follow-up for RCC.

## Conclusions

Precise preoperative diagnosis of the tumor histology and the PAP enabled sufficient preoperative simulations. CP was successfully performed in type 3A PAP for metastatic RCC, avoiding additional resections of the aberrant parenchyma. Intraoperative ultrasound was useful not only to identify the MPD, but also to minimize parenchymal resection, maintaining oncological safety. CP is still a challenging procedure in terms of POPF.

## Abbreviations

CHA: Common hepatic artery
CP: Central pancreatectomy
CT: Computed tomography
DP: Distal pancreatectomy
EUS-FNA: Endoscopic ultrasound-guided fine-needle aspiration
FDG-PET: ^18^F-fluorodeoxyglucose-positron emission tomography
GDA: Gastroduodenal artery
HCC: Hepatocellular carcinoma
MHV: Middle hepatic vein
MPD: Main pancreatic duct
NOS: Not otherwise specified
PAP: Portal annular pancreas
PD: Pancreaticoduodenectomy
PJ: Pancreatojejunostomy
PMet: Pancreatic metastasis
POPF: Postoperative pancreatic fistula
PV: Portal vein
RCC: Renal cell carcinoma
RHV: Right hepatic vein
SMV: Superior mesenteric vein
SPV: Splenic vein
T: Tumor
